# Prison Misconduct and the Use of Alternative Resolutions by Correctional Officers in Therapeutic Communities and Other Custody Units

**DOI:** 10.1177/0306624X211049196

**Published:** 2021-10-09

**Authors:** Michael Weinrath, Caroline Tess, Erika Willows

**Affiliations:** 1 University of Winnipeg, MB, Canada

**Keywords:** correctional officer discretion, diversion, mediation, therapeutic community, institutional misconduct, prisoner misconduct

## Abstract

This mixed methods study uses official records and interviews with inmates and staff to compare misconduct in therapeutic communities (TC’s) and the use of alternative resolutions (in lieu of formal charges by correctional officers) to other prison units. Prisoner misconduct has been studied using individual self-reports or aggregate prison rates, but unit level differences between TC’s and other prison wings are often overlooked. Restorative justice and diversion approaches are much studied in the community corrections literature but correctional officer use of alternatives to charging, such as mediation, is not well understood. The study examines differences in prisoner behavior by unit function by comparing misconduct over a 24 month period in therapeutic communities to general population, worker, protective custody, mental health, and high risk units. Study findings show lower misconduct in TC’s, including more serious misconduct such as fights. Furthermore, a significant proportion of overall charges were diverted into alternative resolution (AR)s, particularly within therapeutic communities. Interviewees reported a different approach taken in the TC toward discipline with a greater use of interaction, informal warnings, and application of AR, as opposed to formal charges. Future research is recommended using qualitative research strategies to appraise the alternative resolution decision process and prisoner-staff perceptions of discipline.

This paper investigates therapeutic community (TC) membership, inmate-correctional officer relations, and their potential influence on prison misconduct and its management. Rates of misconduct between TC’s, general population and other prison units are examined, but unique to this study is assessment of correctional officer use of alternative resolutions (AR), or mediation by staff to deal with inmate misconduct. The mixed methods approach taken here uses quantitative official records data of formal charges and AR, then complements this with qualitative interview data on TC inmate-staff relations.

This study seeks to make several contributions to the research literature. First, studies of TC’s assume better inmate-staff relations but assert this through formal outcomes like misconduct, rather than survey, observation, or depth interviews (Bouffard et al., 2003). Secondly, research reported here will add to a paucity of studies on the use of alternative sanctions or mediation in lieu of formal charging by correctional officers. Considerable attention has been paid to efforts at increasing the use of restorative justice (RJ) type diversions in the community but there has been little consideration of the use of RJ or other mediation systems in correctional institutions. This is important to study because misconduct outcomes for formal charges are more likely to result in disciplinary segregation placement, a 23 hour lock-up situation which often results in higher rates of prisoner distress (Sapers, 2015; Shames, 2015) and an increased probability of recidivism (Butler et al., 2020). Finally, examining prison misconduct by comparing correctional center units provides a contrast to an overreliance on the influence of individual attributes (age, gender, prior record) and aggregate prison characteristics (high security, more coercive, vs. treatment-oriented facilities) to explain variation in offender misbehavior (Randol & Campbell, 2017; Steiner et al., 2014; Worrall & Morris, 2011).

## Correctional Officer Discretion

A fundamental challenge facing corrections administrators is the day to day management of prisoners. Correctional agencies aim to operate institutions in a manner that ensures a safe, secure environment for prisoners and staff, but offender compliance can be a problem given the restrictions and conflict inherent in everyday prison life (Bottoms, 1999; Sykes, 1956). Correctional officers are key to prisoner management and employ a variety of strategies to gain offenders’ cooperation, including the use of discretion in enforcing rules (Weinrath, 2016; Crawley, 2004; Liebling et al., 2010). A subject of contention, however, is to what extent correctional staff should use more formal, coercive measures to respond to prisoner misbehavior, or try to determine other methods to manage them (Colvin, 2000; Day et al., 2015).

A recent focus in the literature has been the importance of correctional officer inmate relations and their influence on misconduct. Correctional officers face a daunting task, as they must enforce rule compliance with individuals who are involuntarily placed under their supervision (Bottoms, 1999). They cannot enforce every rule and have considerable discretion in the decision to charge or not charge prisoners. Obtaining inmate compliance is an important facet of the correctional officer function and has merited considerable study (Hulley et al., 2011; Kolind, 2015; Liebling et al., 2010; Sykes, 1956). It is critical to consider because institutional charges have significant consequences: prisoner segregation in restrictive housing can be required while awaiting a hearing even for minor violations. Overuse can give credence to more general criticisms of prisons as unnecessarily coercive (Butler et al., 2020; Shames, 2015). For example, critics cite overuse of segregation or different forms of isolated detention (or what is now more often called “restrictive confinement”) as an example of correctional officers and administrators being too quick to use intimidating measures to deal with prisoner misconduct (Hastings et al., 2015; Sapers, 2015; Shames, 2015).

As an alternative, correctional officers can use their own informal authority to encourage more prosocial behavior or deescalate aggression, the so-called “tactics of talk” (Liebling et al., 2010). In North America, direct supervision was introduced in the 1970s, and touted as means to improve inmate relations by focusing on building relationships with offenders (Wener, 2006). More recently, it has been recommended that correctional officers taking a direct supervision approach incorporate procedural justice processes in their communication and behavior, giving inmates voice, being respectful, consistent in their rules and impartial in dealing with violations (Weinrath & Coles, 2003; Liebling & Arnold, 2005). Inmate perceptions of procedurally just behaviors by correctional officers is associated with lower misconduct rates (Beijersbergen et al., 2015; Bosma et al., 2020).

## Related Literature on Informal Alternatives

Before considering correctional officer discretion further, it is profitable to consider related police literature, which offers a more substantial body of research. Of particular interest is police discretion on the use of diversion or restorative justice (RJ) measures as an alternative to formal criminal charges (Bazemore & Griffiths, 2003; Wemmers, 2002). At the police level, RJ has been touted as a method to provide a different response to crime, moving away from adversarial systems of determining guilt and punishment, and seeking instead to repair harms, using dialog to mediate disputes between parties. Ideally, use of RJ would contribute to lower reoffence, higher victim, offender and public satisfaction with the justice system and a less punitive and coercive justice system. RJ has been available in many parts of the Western world for years but has yet to exert a significant impact on criminal justice actors (Wood, 2015). This is not surprising given that extensive community based criminal justice research on the use of diversion programs or RJ systems generally finds that police are reluctant to use diversion except for minor crime or first offenders (Abramson, 2003; Crocker, 2013).

Within a prison setting, Butler and Maruna (2016) reported on the negative reactions of offenders to UK prison disciplinary systems, which are still operated by custodial staff rather than independent adjudicators. In their semi-structured interviews with 31 prisoners they found considerable feelings of unfairness directed toward the disciplinary tribunals that heard misconduct charges in four British prisons, because correctional administrators ran them and always took the word of the correctional officers over theirs. Staff interviewed as part of the study, however, argued that administrative paperwork made charging so burdensome they would not charge for minor transgressions or instead use their incentive system (positive vs. negative reports) to manage misbehavior. The authors argued that RJ processes could do much to create a greater sense of legitimacy by prisoners toward prison administration and reduce the use of punitive sanctions such as segregation (Butler & Maruna, 2016). Sanctions related to informal restorative practices would be viewed as more legitimate than reports perceived as capricious by inmates.

The restorative justice approach recommended by Butler and Maruna is relevant because of a shift to mediation type policies implemented in correctional systems over the past 20 years. Besides informally deciding not to charge, correctional staff have other options to the official processing of infractions (Correctional Service of Canada, n.d.; Federal Bureau of Prisons, 2011; Manitoba Correctional Services Act, 2014; Ontario Ministry of Community Safety and Correctional Services Act, 2018). An available strategy in many correctional jurisdictions is the use of informal or alternative resolutions in the event of prisoner misconduct. Rather than formally charging and then possibly segregating prisoners, policy in correctional settings such as Canada and the US allow for resolution of misconduct by correctional staff and prisoners outside of the disciplinary process Going by titles such as “alternative resolution” or “informal resolution,” these processes are generally not as structured, involve less serious misconduct and incur lesser penalties, including suspended sentences or warnings, and can replicate some of the features of restorative justice (Butler & Maruna, 2016). While these procedures have been available for some time, there is scant research available. This is surprising, given the concern over the overuse, underuse or even inconsistent application of aggressive measures to manage individual behavior, particularly in correctional institutions (Butler & Maruna, 2016; Colvin, 2000; Day et al., 2015; DiIulio, 1987).

For this study, legislation and policy reviewed in US and Canadian federal and provincial facilities showed that informal procedures were vaguely outlined. Sometimes legislation would suggest trying to work out an informal resolution by not charging, other times it would reference use of informal resolution procedures (Correctional Service of Canada, n.d.; Federal Bureau of Prisons, 2011; Manitoba Correctional Services Act, 2014; Ontario Ministry of Community Safety and Correctional Services Act, 2018). The provincial correctional center where this study was performed used sit-down mediation sessions where, after discussion and acceptance of responsibility, an apology, restriction of some minor privilege or performance of community work (often unit cleaning duties) would be assigned rather than a formal charge.

## Prison Units, Therapeutic Communities, and Misconduct

Better environments (social climates) are thought to be one way to improve prison life for offenders and staff, leading to safer correctional facilities and more opportunities for rehabilitation (Auty & Liebling, 2020; Bosma et al., 2020; Harding, 2014). Building from the organizational psychology literature, the prison environment is described by scholars as an interaction of physical, social, and emotional conditions as perceived by residents (Liebling & Arnold, 2005; Moos, 1975). Prison environment studies have generally been done at the macro level by comparing two or more prisons at a time using quantitative methodology or have focused on a single institution. Generalizing about an homogenous prison environment can seem curious to anyone who has worked in a correctional institution. An individual prison might have an overall culture that distinguishes it from other facilities (Day et al., 2011; Liebling & Arnold, 2005; Sparks et al., 1996), but day to day experiences within a prison can vary significantly depending on where a prisoner (or staff) is housed or visiting.

Correctional facilities are not unidimensional in purpose and this becomes clear when a prison is broken down by the functions and purposes of different units or wings and designated areas such as protective custody, program units, designated work sites, and visiting. These areas are often physically segregated from other parts of the institution, lessening the influence of an often negative prisoner subculture (Kolind, 2015; Mears et al., 2013) and, to a degree, correctional officer emphases on social distancing from offenders (Crawley, 2004; Farkas, 2000). The differing regimes and informal rules in these units and distinct areas can facilitate more distinctive behaviors and prisoner-prisoner and prisoner-staff relationships (Kolind, 2015; Schalast & Laan, 2017).

Therapeutic communities (TC’s) present as an almost ideal type of mechanism to promote a positive prison environment at the unit level. TC’s in prisons are separated physically from other areas and provide structure and an interactive milieu for offenders to support each other, as well as encouraging interaction with staff (De Leon, 2000). Their purpose is to use the TC milieu, or “community as method” approach to facilitate offender change. Research on TC’s supports the notion that prisoners participating will differ in behavior relative to other areas of a prison such as the general population. Using a variety of prisoner samples, custodial settings and outcomes, research consistently demonstrates that therapeutic communities show lower rates of institutional misconduct for a variety of misbehavior (Auty et al., 2017). Dietz et al. (2003) found less violence and general institutional infractions and grievance rates among TC participants compared to units in the same prison, noting that the two groups were roughly equivalent in time served, age and prior criminality. Prendergast et al. (2001) and Deitch et al. (2004) found lower rates of violent and other infractions between TC participants and general population prisoners in comparisons between TC and non-TC prisons. In a more recent large-scale study, Taylor et al. (2019) found lower misconduct rates by TC participants over a 3 year period, and lower misconduct rates over all by prisoners who participated in other programs compared to non-attenders.

## Staff Relations, Therapeutic Communities, and Prison Places

Staff behavior can also be impacted by regime and expectations. For example, investigators have found that prisoners in protective custody felt more supported by staff (Casey et al., 2016). Kolind (2015) found that Dutch correctional officers were supportive of drug treatment wings or units, and often did not charge participants for minor drug use or possession because they did not wish to cause disruption in the program area. Officers felt that they gained more over-all positive behavior from offenders for letting some minor drug offenses go, while the resentment caused by a charge would create more problems within the unit. There are limits to this approach, however, and challenges for correctional officers in successfully cultivating informal authority. In her British study, Liebling (2011) found that failure by correctional officers to enforce rules led to more predation by some prisoners against others. In comparing two prison wings, she found that on one unit where staff were too focused on accommodating a few influential prisoners and ignored rules, non-enforcement led to more inmate-inmate victimization.

Research on differences in staff behavior toward prisoners by unit function is most robust in TC’s. Staff working in TC’s are expected to undergo training and support the goals of the program (De Leon, 2000). Given this, therapeutic communities present as having an environment that would discourage restrictive sanctions by correctional officers compared to other living areas within a correctional setting. Indeed, better staff outcomes have also been achieved in TC’s such as less correctional officer absenteeism, less inmate assaults on staff, less injuries by staff if assaulted, more concern for offender well-being and higher ratings by staff of the prison environment (Deitch et al., 2004; Prendergast et al., 2001). In addition, policy makers that find cost benefit support for the utility of using therapeutic communities relate that much of this comes from positive staff impacts. In their economic analysis of a prison TC, Zhang et al. (2009) found substantial savings from lower administrative costs resulting from less serious incidents (e.g., fewer prisoner-prisoner, prisoner-staff assaults) and less prisoner grievances in TC’s compared to traditional correctional units. The authors further found savings through less staff overtime, physical damage from serious incidents and reduced medical costs.

In sum, residing in different areas within a correctional institution can result in different prisoner and staff behavior. Research on this is particularly strong with respect to therapeutic communities.

## Research Questions

This research examines prison misconduct and the use of alternative resolutions in a TC versus formal charges by correctional officers within a correctional institution and compares misconduct and utilization of alternative resolutions with other prison units. A secondary interest is qualitatively assessing inmate-correctional officer interactions in the TC and contribution of these relationships to application of alternative resolutions.

Hypothesis 1: Therapeutic communities will report less misconduct than other prison units.
Hypothesis 2: Correctional officers in therapeutic communities will be more likely to use alternative resolutions than staff in other prison units.
Hypothesis 3: TC inmates and assigned correctional officers will report better relations and a different approach to discipline in TC units compared to other prison units.

## Study Setting

The fictitiously named Provincial Correctional Center (PCC) is a Canadian provincial multi-purpose medium/minimum security facility housing pre-trial detention and sentenced provincial offenders serving up to 2 years. PCC is over 90 years old and features dormitories and old linear style units, but it also has much newer direct supervision podular units built in the early 2000s, using a high visibility style of architecture. PCC is rated at around 550 prisoner capacity but regularly held over 800 prisoners for a number of years prior to and after this study. Protective custody, mental health and high risk/gang offenders were housed in the newer units. The unit breakdown below provides the maximum occupancy (beds) but relies on double-bunking. The prison was always full during the study period. PCC received longer-term remand and sentenced prisoners sent out from admitting and pretrial detention facilities daily, allowing them to maximize occupancy. All prisoners were double bunked as well due to the crowding.

The unit descriptions are as follows: General population, GP, (*n* = 298): The linear style units in the old main building at PCC held general population inmates. Individual double-bunked units housed 28 inmates. Kitchen workers, KW (*n* = 64): Two dorm style units contained mostly kitchen workers, a group of prisoners who had displayed a good work ethic and above average behavior within the institution. As one of the few paid positions at PCC, the kitchen worker assignment was a popular placement. Protective custody, PC (*n* = 94): prisoners housed in these units either requested placement or were put there due to threats, assaults or serious incompatibility problems, usually from general population units. Prisoners could request placement, staff could petition for their placement because of concern for their safety, and sometimes PC would be recommended by senior prisoners on a unit (“Move inmate A because someone will hurt them”). Mental health, MH, (*n* = 90): prisoners with demonstrated mental health conditions were housed within these units. There is considerable variation in mental health challenges, as offenders ranged from those with limited cognitive functioning to those with DSM conditions who received regular medication. While the MH unit was operated in a more benign fashion it was not a TC approach. High Risk/Gang, HR (*n* = 127): These units consisted of higher risk prisoners deemed a threat to others, entire gangs and those housed in segregation. Sometimes two gangs would end up doing “shifts” in the same unit common area to avoid contact with rivals (Gang A out from cells in the morning, Gang B in the afternoon).

Two separate buildings with dorm style areas were used to accommodate the TC’s. The PCC modified therapeutic community for drug users began operation in October 2012. Although a modified program it followed a fundamental structure similar to the program outlined by De Leon (2000). The program focused on drug addicted offenders and admitted both pre-trial detention and sentenced prisoners in units TC1 and TC2. Participants worked their way through a four-phase program. New recruits were housed in TC1 (*n* = 84), while participants who achieved advanced through phases 1 and 2 usually moved to TC2 (*n* = 72). The program provides structure through work and program routines and prisoners have assigned responsibilities and leadership roles. In addition to self-study the program requires completion of modular 1 or 2 week sit down group programs to progress. The “community as method” approach to behavior change was used and participants were expected to encourage each other and attend regular group meetings. Rules were strict on violence and even horseplay. While correctional officers in all parts of PCC were supposed to engage in the direct supervision style^1^ of interactive offender management, correctional staff recruited to work in the therapeutic units received TC training and were expected to emphasize interpersonal relations even further and support the goals of the TC.

## Methodology

The study used official records to compare disciplinary charge rates, alternative resolutions, forced disciplinary and incompatibility moves per unit bed by prison unit over a 2-year period. The comparability of the units was first established using an ANOVA comparison of static risk classifications. Open-ended question responses for 20 TC inmates and 14 correctional staff were used to assess unit relationships. The study used a concurrent mixed methods design, with the qualitative interviews embedded in the quantitative assessment of official misconduct. Mixed methods ideally sees researchers create more comprehensive research designs that can ultimately generate more insights into phenomena and improve validity and reliability of findings (see Hesse-Biber & Johnson, 2015). The qualitative interviews of inmates and staff were used to explore uses of AR but analysis focused on confirming the association between inmate-staff relations in a TC regime and correctional officer use of AR.

### Measuring Institutional Misconduct

Corrections head office gave researchers PCC charge and incident data, broken down by unit type, from October 1st, 2012 to September 29, 2014, a 24 month period. *General Institutional Misconduct* was a general category that captured most violations (fighting/assault, disrespect to staff, disobey order, counsels offense, disturbance, gang activity, violates rule, unauthorized telephone use, and possession of contraband). Rates were calculated for each category by dividing raw number totals by the number of beds in each unit (e.g., dorm units total 100 beds, experience 20 rule violations over a 2 year period, misconduct rate = rule violations/beds, or 20/100 = 0.20). As is the case in most prison research, exact data was unavailable on bed occupancy over the 2-year period, but in a check of the population lists cited earlier, over a 12 month period the units generally ran at or just slightly over capacity with double-bunking the norm.

The data collected was from official records, and hence likely misses a fair proportion of misconduct that goes unreported by prisoners or unrecorded by correctional officers using discretion (Cooley, 1993; Steiner & Wooldredge, 2014). In their key comparison study of official versus self-report misconduct, Steiner and Wooldredge (2014) concluded both methods showed validity and recommended longer periods of study, something that was accomplished with use of the 24-month period in this study.

While a general misconduct measure provides important information on general prisoner misbehavior, two additional measures were created to categorize more serious institutional responses to misconduct, and to assess whether or not some prison units provided more collegial environments. *Forced Moves to Segregation* counted instances of prisoners being moved to segregation because of being charged for a disciplinary infraction. These moves were notable because they involved situations of fighting or where an incident occurred that showed the prisoner posed a threat on the unit to staff or other offenders. Being forced to move to segregation is a serious restriction of liberty, as this involves being placed on 23 hour lockup with limited or no privileges, pending a disciplinary board hearing. *Forced Moves due to Incompatibility* was created by counting prisoner moves to another unit or protective custody due to incompatibility (moves initiated for prisoner safety). Moves due to incompatibility are a result of a perceived threat to one prisoner posed by others, and have complicated day to day prison life in federal and Canadian correctional institutions (Weinrath, 2016; Sapers, 2012). As noted earlier, moves can be initiated by individual prisoners, staff, or senior prisoners on a unit who view an individual as likely to be assaulted.

The two disciplinary and incompatible forced moves were combined into one *Forced Moves* variable.

To assess the willingness of staff to use discretion and mediate conflicts, *Alternative Resolutions* was estimated using automated official records data on charges, broken down into formal charge and alternative resolutions. An alternative resolution would only be counted if mediation was agreed to by staff and prisoners.

### Qualitative Data

As part of another study, 20 senior TC inmates (third or fourth Phase) and 14 TC staff were purposively sampled for semi-structured interviews. A focus of the original study was inmate-correctional officer relations in the TC. Informed consent was obtained and one on one interviews with study subjects were conducted in a private office within the TC. One researcher interviewed 17 inmates, another interviewed 3, the principal investigator (PI) reviewed responses for both and found consistency in recordings (i.e., no concerns for inter-rater reliability). The PI interviewed all 14 staff. Inmate open-ended survey questions directly addressed staff efforts to manage compliance: *How do staff members confront inappropriate* behaviors*? Can you tell me how staff discipline people in the TC? Do you agree with the methods used?* For staff, questions were more indirect in assessing the relationships, including: *Overall, how would you describe your professional relationship with the inmates in the TC?* Comparison with relations in other units was appraised by asking: *How would you rate working at the TC compared to other units at PCC?* Inmate relations were also brought up indirectly by responses to questions about *What are the strengths, weakness of the TC?*

Recruitment and interview protocols were reviewed and approved by the principal investigator’s University Research and Ethics Board (REB).

### Analysis Plan

A retrospective analysis of official records compared different prison units on misconduct outcomes, forced moves and on staff use of alternative measures in place of disciplinary charges. The equivalency of general risk between most PCC housing units was established using the ISA risk instrument, means comparison, and Bonferroni correction (see below). A one-way ANOVA was then used to first compare misconduct mean rates from October 2012 to September 2014 (24 months) between the TC’s and various units in the prison, again applying a Bonferroni procedure to test for statistically significant group differences. A mean misconduct rate per prison bed was estimated using the average prisoner occupancy and then dividing it by the total number of misconduct charges (e.g., high risk units average 127 prisoners, 171 charges laid, charges divided by prisoners equals around 1.35).

Forced prisoner segregation and incompatible moves were combined to represent a general measure of how well prisoners can get along and estimated in a similar fashion to misconduct: a rate per unit bed count was established and then compared, and again utilizing ANOVA to assess significance. Finally, the proportion of misconduct charges in each unit that were successfully diverted into alternative measures were contrasted using column percentages, and a chi square test statistic calculated for significance testing.

Qualitative responses to open-ended questions were recorded by hand verbatim and then entered into a word-processing file for analysis. Descriptions of the inmate discipline approach in the TC and use of alternative resolutions by correctional staff were the focus of analysis. In reviewing written text, it was felt that saturation in findings was actually achieved prior to all subjects being interviewed.

## Determining the Comparability of Prison Spaces

The study compared misconduct rates with different outcomes across different units by their function such as TC, GP, KW, PC, MH, and HR. The TC units used in this study did not have prisoners randomly assigned, so selection bias is a potential design weakness. Perhaps only lower risk offenders with short or non-existent criminal histories made up the TC groups, while higher risk prisoners were housed elsewhere? This might explain variation in disciplinary infractions and render comparisons invalid. To partially control for this, security risk ratings by unit were used. If analysis showed that TC participants had risk scores similar to other units, there was a reasonable expectation of equivalency, at least for potential misconduct. Risk scale scores are typically strong predictors of prisoner misbehavior, not surprising as scale items often comprise known predictors of misconduct and reoffence such as age, prior criminal history and institutional behavior (Gendreau et al., 1997; Steiner et al., 2014; Worrall & Morris, 2011).

Risk scores at PCC were determined by the Institutional Security Assessment (ISA), a ten item instrument comprised of static items such as age, severity of current offense, severity of prior criminal history, past institutional misconduct, parole history, and youth record. The ISA has been previously validated and was found effective in predicting future prison misconduct and reoffence (Riccirdelli et al., 20xx). It included items that assess the three prime correlates of misconduct: age, prior criminal history, and prior institutional misconduct.

PCC is a medium/minimum security institution so there were no maximum security units, but there was a high risk/gang area that included some segregation cells. Prisoners were placed in a general population unit unless assigned to the kitchen workers, protective custody, mental health or therapeutic community units. Like any correctional institution, the PCC prisoner population changes were ongoing. Precise daily averages were not available, so twice monthly on Thursday or Fridays researchers were allowed to access a prisoner population list, broken down by unit, giving security status via ISA score (ranges of 0–16). Averages presented in Table 1 were calculated over a 24 month period (48 data points) from October 2012 to September 2014 and then compared using one-way ANOVA with a Bonferroni adjustment. As can be observed from the individual unit population numbers, PCC was generally run overcapacity and thus individual units were full during the study period. No time periods were experienced where units appeared to be below or above capacity, give or take one or two prisoners. The correctional system and PCC administration were efficient at keeping all units full, although this did mean double bunking. The TCs at PCC did not have fixed start dates, much of the program consisted of work routines, meetings, interaction, and self-study, with week-long workshops offered periodically to allow progress to the next phase. Thus the program was able to allow constant entry, making it easier to maintain units at full occupancy.

**Table 1.** Mean Institutional Security Assessment (ISA) Rating by Prison Unit^a^ for October 2012 to September 2014.

	Unit	Prisoner	ISA mean	*SD*	Units in each row noted by numbers are significantly different at *p < .05.^b^
1	GP	14,000	5.98	2.46	2, 5
2	KW	3,080	5.45	2.36	1, 3, 4, 5, 6, 7
3	PC	4,500	5.96	2.64	2, 5
4	MH	4,400	5.93	2.38	2, 5
5	HR	5,096	6.55	2.53	1, 2, 3, 4, 6, 7
6	TC1	4,012	5.88	2.12	2, 5
7	TC2	3,426	5.96	2.25	2, 5
	Total *N*	38,514	5.99	2.43	
		*Bonferroni correction* *F* = 74.11***, 6 *df*.			

*Note*. ISA range 0 to 16. ***p < .000.

GP = general population units; KC = kitchen worker units; PC = protective custody units; MH = mental health units; HR = high risk/gang units; TC1 = therapeutic community unit; TC2 = therapeutic community for advanced Lanyards.

a Prisoner *N* summed from twice monthly snapshots October 2012 to September 2014 (unit total × 48). Prisoner ISA mean score based on 10 items (classification is 0–5 low, 6–10 medium, 11 up high). Prisoner *N* summed from twice monthly snapshots October 2012 to September 2014.

b For example, in first row GP unit (1) mean of 5.98 is different from (2) Kitchen Workers 5.45 and (5) High Risk unit mean of 6.55.

The average ISA score was 5.99 (*SD* = 2.25), right around the score of “6” that results in medium security status (Table 1). As might be expected, the HR unit had the highest mean score (6.55) while the tightly screened KW unit had the lowest (5.45). Observed differences by the two TC units, TC1 and TC2, were statistically significant compared to the HR (lower risk) and the KW units (higher risk). The TC’s were similar in risk to GP, PC, and MH units. These five units ranged narrowly in mean risk scores, going from 5.88 (TC1) to a high of 5.98 (GP).

It was expected that the elevated ISA risk scores would be likely to correlate with higher misconduct rates for the HR unit. Conversely, fewer infractions in the KW unit were anticipated because risk levels there were relatively low.

## Results

### Institutional Misconduct by Unit

Over the study’s 2 year period, there were clear differences in misconduct by unit (*F* = 74.11, 6 *df*, ***p < .001). Not surprisingly, the HR units had the highest rate of misbehavior (1.35 per prisoner), and the observed mean differences from other areas of PCC were all statistically significant, except for MH (Table 2). The MH unit average was also quite high (1.27) and distinct from other units except for HR. The TC2 unit had the lowest misconduct rate, and was significantly different from all other units. The GP average was third highest (0.98) with statistically significant differences from all but the PC units. The PC unit mean of 0.94 was statistically significant when differences were estimated against the HR, MH and TC2 units.

**Table 2.** Misconduct Rate per Unit Beds by Prison Unit for October 2012 to September 2014.^a^

	Unit	Prisoner	Misconduct charges	Mean per unit beds	*SD*	Units in each row noted by numbers are significantly different at *p < .05.^b^
1	GP	298	292	0.98	0.14	2, 4, 5, 6, 7
2	KW	64	50	0.78	0.42	1, 4, 5, 7
3	PC	94	88	0.94	0.25	1, 2, 7
4	MH	90	114	1.27	0.45	1, 2, 3, 6, 7
5	HR	127	171	1.35	0.48	1, 2, 3, 6, 7
6	TC1	84	69	0.82	0.39	1, 4, 5, 7
7	TC2	72	31	0.43	0.50	1, 2, 3, 4, 5, 6
	Total	829	815	0.98	0.43	
			Bonferroni correction *F* = 70.74***, 6 *df*.			

***p < .000.

a Prison overcrowded and mostly at capacity during October 2012 to September 2014.

b For example, in first row GP unit (1) mean of 0.98 is different from (2) kitchen workers 0.78, as well as other units listed as 3, 4, 5, 6, and 7.

The KW unit with carefully screened inmates had the lowest ISA scores, so it is not surprising that they recorded the second lowest misconduct rate (0.78). The mean differences for KW compared to HR, MH, GP, and TC2 units were statistically significant but not for KW compared to PC and TC1 units. The lowest misconduct rate by far was TC2 (0.43), the unit, holding mostly senior therapeutic community members; despite having similar ISA risk scores to most other units, TC2 showed much less disruptive behavior over the 2-year study period.

The TC1 score of 0.82 was almost twice the TC2 rate but still third lowest among all prison units. Recall that TC1 had a similar ISA risk profile to other units, so the lower rate of disciplinary infractions most likely speaks to the favorable impact of the TC environment. The lower rate in TC2 compared to TC1 was expected, given that the long-term TC members have more actively participated and worked their way from entry level to higher lanyard status to get into TC2. Furthermore, TC1 houses program newcomers, some of whom may not stick with the program, or remain in the program but may be released prior to moving on, or just not be successful in progressing into TC2.

Results for general misconduct support hypothesis 1, that TC’s would have lower misconduct rates than other units. However, the low rates by the KW unit illustrates how selection effects (i.e., screening low risk inmates into certain prison wings) can also influence misconduct rates within an institution.

### Forced Disciplinary and Incompatible Moves by Unit

During the 24-month study period at PCC the number of forced moves per prisoner was 153 for incompatibility, 118 for disciplinary moves, a rate of 0.33 per prisoner bed (Table 3). There were no incompatible moves for either TC1 or TC2 during the 24 months covered. For disciplinary moves, there were only 2 for TC2 and 15 for TC1, the second lowest aggregate number amongst PCC units. The absence of these types of coerced moves was quite remarkable and suggests a substantive influence of TC environment in promoting more collegial behavior amongst residents, or at least in discouraging aggressive behavior. The estimated overall forced moves rate of 0.18 for TC1 and 0.03 for TC2 were the lowest amongst all the units assessed.

**Table 3.** Unit Type by Forced Prisoner Moves Due to Disciplinary Charge or Incompatibility October 2012 to September 2014.

	Unit	Prisoner	Forced move incompatible	Forced move disciplinary	Forced moves total	Mean forced move per unit beds	*SD*	Mean difference from other units significant at *p < .05
1	GP	298	108	30	138	0.46	0.50	2, 3, 5, 6, 7
2	KW	64	8	9	17	0.27	0.45	1, 4, 7
3	PC	94	11	15	26	0.28	0.28	1, 4, 7
4	MH	90	14	33	47	0.52	0.50	3, 5, 6, 7
5	HR	127	12	14	26	0.21	0.41	1, 4
6	TC1	72	0	15	15	0.18	0.39	1, 4
7	TC2	84	0	2	2	0.03	0.17	1, 2, 3, 4
	Total	829	153	118	271	0.33	0.47	
			F = 16.516***, 6 *df*.					

***p < .000.

Combining the disciplinary with incompatible moves into a “forced moves” indicator, the most frequent rate was found for the MH (0.52) and GP (0.46) units, and their differences were statistically significant when compared to all other units, but not from each other. TC2 had the lowest rate by far (0.03) and its mean difference was statistically significant from all other units. The forced move rates for HR, PC, and KW clustered from 0.21 to 0.28, and were different at *p < .05 from the GP, MH, and TC2 units. TC1 results were in the predicted direction and had the second lowest rate of forced moves (0.18) but displayed statistically significant differences only from the GP and MH units. The aggregate or rate differences in many forced move comparisons were substantial, but the variability (as indicated by the high variance ratio of standard deviation to mean), made it difficult to demonstrate stable, statistically significant differences.^2^

As expected, TC1 and TC2 showed a lower probability of ordering someone off their unit due to incompatibility, suggesting a greater ability to get along by TC prisoners. The lack of prisoners being placed in disciplinary segregation is likely due to better overall behavior, but also perhaps a greater likelihood of TC officers being less likely to require segregation when laying a formal charge (i.e., better prisoner-officer relations).

The lack of coerced movement observed in the TC’s provides further support for hypothesis 1.

### Alternative Resolutions

The overall rate of charges that resulted in alternative resolutions was 35.5%, or slightly over one in three charges indicating a substantial use at PCC (Table 4). A single 7 × 2 cross-tab was calculated, and differences were statistically significant (χ^2^ = 70.334, 6 *df*, ***p < .*000*). The lowest use of alternative resolutions was in the high risk area, only 15.8% of the time. High risk prisoners are likely not great candidates for alternative resolutions, but they would also have to contend with the lack of available alternatives—HR were already restricted in their movements and did not have many privileges to lose. The GP, MH, and PC rates were all around 33%, or a third of the time. Again, the TC units did much better than most of the other prison units. TC1 saw alternative resolutions used in about one out of every two cases (47.8%). At 64.5%, TC2 shows that almost two-thirds of disciplinary charges were dealt with by alternative resolutions. While the outcomes for TC’s are laudable, the highest proportion of alternative resolutions was observed in the KW unit, 70%. Clearly, prisoner-staff relations were favorable on these units, but KW does screen and take lower risk prisoners, so more positive mediation was expected.

**Table 4.** Misconduct Charges by Unit Type and by Proportion Diverted Into Alternate Resolutions.

Unit	Misconduct charges	Alternative resolutions	Percentage of charges
GP	292	101	34.6
KW	50	35	70.0
PC	88	31	35.2
MH	113	42	36.8
HR	171	27	15.8
TC1	69	33	47.8
TC2	31	20	64.5
	814	289	35.5
	χ^2^ = 70.224***, 6 *df*.		

***p < .000.

Because chi-square gives an aggregated estimate of statistical significance and does not compare units directly, separate 2 × 2 cross-tabs were run with the two TC units individually against other units (not shown, results available on request). TC1 showed a smaller to moderately greater likelihood of working out more alternative resolutions, with differences ranging from 12.6% to 32.0% in the predicted direction with other units. These differences were statistically significant contrasted in 2 × 2 tables with the HR, GP, and KW but not PC, MH, or TC2 units. In 2 × 2 table comparisons, TC2 also showed a greater likelihood of mediating charges, 16.7% to 48.7%, and these variations between units were statistically significant for HR, GP, MH, and PC. TC2 was 5.5% less likely to use alternative resolutions than KW, but this difference was not significant (Not shown, available on request).

Results from the comparison of alternate resolution use provides support for hypothesis 2.

### Inmate and Staff Interview Data

When asked about the general disciplinary process, inmates described methods of confronting behavior. In addition to program related punishments (e.g., being set back from Phase III to Phase II, expelled from the program) inmates identified program informal and progressive discipline being used:

*“They’ll pull you aside, in the office, try not to make it too obvious. They get training in progressive discipline I think, give warnings. Ask if you are having a bad day.”*

*“Reprimands, AR’s. Spoken to with letting them know and giving them an option how to correct it. AR means loss of privilege. Loss of canteen, loss of BBQ, clean up a certain area of the unit.”*

*“They pull them out and confront them and ask them what’s going on, if they’re having a bad day or something. Pull us in the office and talk it out.”*

Inmates generally agreed with the compliance measures used by staff in the TC. They believed that rules and their enforcement were necessary for the security of the unit and the successful operation of the TC program. They commented on the different relationship with staff compared to the general units, observing that there was more two way dialog:

*“Staff here talk more, listen more than in the main building.”*

*“Its easier here to have a conversation with staff.”*

The staff viewed the relations with inmates as markedly different in the TC compared to other units in the prison. They believed that they interacted much more frequently than in general population units. They felt that the central principles of direct supervision (e.g., stronger relationships, more inmate-staff communication to resolve programs) were being achieved more consistently within the TC setting. They attributed this to the TC environment through its emphasis on the “community as method,” as well as their own training and orientation. The TC program impacted inmates favorably, making them more likely to talk to staff in the TC, paving the way for stronger relationships to develop than in other units in the prison.

Results from inmate and staff interviews were consistent in supporting hypothesis 3.

## Discussion and Conclusion

This mixed methods study found support for all three hypotheses. First, as expected, the therapeutic communities evinced lower rates of misconduct than other units in the prison research site (hypothesis 1). Over a 2-year period despite presenting an equivalent risk profile to other units, prisoners participating in the TC’s averaged less general misconducts. This difference persisted when more serious misbehavior such as forced moves for violence or incompatibility were considered. Correctional staff did not have to move prisoners off TC’s as frequently as general population or other units due to serious fighting, assaults, or threats. Removing offenders from units because of physical threat reflects a reluctance or unwillingness of prisoners to get along with each other. Incompatibility moves were also lowest in the therapeutic communities, indicating that greater tolerance was practiced amongst participants.

Data also supported hypothesis 2. Alternative resolutions, a tactic rarely studied in correctional settings, was used just over a third of the time at PCC, which showed a pattern of widespread staff adoption of this practice. As predicted, therapeutic communities were more likely to mediate disciplinary infractions in this way compared to other parts of the prison. The “community as method” for the prisoners as well as correctional officer training and orientation required by the TC program appeared to have an impact on subsequent behavior of both inmates and staff.

Finally, using a mixed methods approach, interview data with inmates and staff found that they both perceived more positive relations in the TC, and use of more informal measures of discipline, as well as greater recourse to alternative resolutions (Hypothesis 3). The community as method TC approach, as well as staff training clearly contributed to this.

Otherwise, some of the other findings were perhaps not particularly insightful but consistent with the literature: high risk units showed higher rates of misconduct and lower rates of alternative resolution use while the lower risk, carefully selected and incentivized worker units showed lower rates of misconduct. The more negative unit of HR inmates achieved only a low alternative resolution rate of 15.8%, but it reflected at least some effort by staff to work with prisoners designated higher risk, as opposed to a zero-tolerance approach other more punitive security regimes might take. The KW unit use of 70% AR likely speaks to their low-risk status and subsequent good prisoner-staff relations, but it may also indicate reluctance by staff to formally charge and risk losing a good worker.

Lower misconduct results suggest that offenders get along well with each other within the TC environment, but alternative resolution data coupled with qualitative interview findings confirm that a different relationship exists between correctional officers and prisoners within this program setting.

More needs to be done to determine how the TC worked to increase use of alternative resolutions. For example, could features of the TC training be adopted to general orientation training of CO’s to bolster the effectiveness of direct supervision training? How do correctional officers perform if moved from the TC to the general population units? Are they still more likely to utilize alternative measures when not working with TC inmates? The job of a correctional officer is a difficult one, mechanisms that improve their ability to manage inmates are sorely needed (Ricciardelli, 2014).

This study contributes to research indicating lower misconduct rates are associated with TC’s (Auty et al., 2017; Taylor et al., 2019), as well as general research correlating less misbehavior by prisoners in program oriented units (Auty et al., 2017; French & Gendreau, 2006; Randol & Campbell, 2017; Taylor et al., 2019). Findings reported here also provide support for further inquiry into prison units and the impact their functions can have on prisoner behavior. Prison wide surveys and aggregate comparison of official prison records are worthwhile strategies but studies at the unit level can tell us much about prison life, the differences within a prison by area function and point the way to strategies for improving prisoner management. This study provides support for a mixed methods approach to research, at least correlating lower quantitative misconduct outcomes with the presence of more interactive and mediation based management approaches by correctional officers. Interviews with the inmates also unearthed indications of a progressive disciplinary approach being used by staff, providing much more context in our understanding of prison discipline. Generally, quantitative approaches tell us little about how discipline is experienced by prisoners within their custodial setting.

This research provides a springboard for further investigation of correctional officer use of diversionary or mediation programs like alternative resolutions. In this study the widespread adoption of alternative resolutions appeared positive, and its usage in the work unit and TC’s was excellent. There are many features of the TC that make this outcome probable. The emphasis on dialog and planning for progress through the TC’s phases encourages dialog between inmates and staff, and such interaction must lead to stronger relationships. Being involved more directly in the casework processes of the TC program, CO’s themselves have a stake in seeing an inmate work through the phases. Thus use of alternative measures becomes more important to CO’s because formal charges for an inmate mean a delay in the program or even expulsion from the unit.

But many questions remain about the general use of alternative measures. Has the availability of this alternative process resulted in more charges overall (widening net), has it lessened formal charges, has use of segregation declined? What controls are there to ensure fair and impartial use given possible divergent staff views on discretion? Do staff take a restorative justice approach or is alternative resolution something different? Do prisoners see the use of alternative resolutions as a way of making the prison disciplinary process more just or is it viewed as another means to oppress them? These questions are all worthwhile and could be investigated by a variety of official records, survey and qualitative methodologies.

Potential selection bias effects in this study are acknowledged, and this mitigates the strength of findings. TC participants do apply for entrance into the program (motivated for treatment). It is noteworthy, however, that the TC’s in this study report less misconduct despite having high risk prisoners in their programs. Programs like TC’s can serve an important function by providing a constructive experience for prisoners, a platform for at least some offenders to improve themselves (Gendreau & Keyes, 2001) and a method of improving the overall prison environment and lowering conflict between prisoners (Bosma et al., 2020; Liebling & Arnold, 2005).

There are several other limitations to this study. Prison based research accessing official records is subject to common problems associated with use of existing data. As noted earlier, the likelihood of unrecorded or unobserved misconduct means that this study underestimates the amount of misbehavior during the study period (Steiner & Wooldredge, 2014). Charges and forced moves may not have been recorded, or perhaps prisoners were moved off units surreptitiously with no record of conflicts with others. Misconduct was aggregated as a general measure instead of by violent, drug, or property violations, as has been done in other studies. The use of more varied charge outcomes was unfortunately limited by sample size. A case was made for the equivalency of most units by comparing average ISA ratings, but this was based on snapshots over a number of months and was not a true averaging of prisoners in each unit every day over that period. Given the prisoner turnover within provincial facilities, limiting analysis to snapshots contributes to a lack of precision. It is acknowledged that this study falls far short of a randomized controlled trial, yet the turnover of prisoners and challenge of accurate daily counts is a problem plaguing all prison-based research. The use of snapshots and risk data used here is one method of assessing a prisoner population and providing an averaging of risk.

Finally, other qualitative data was lacking that might give more insight into why the TC’s were able to report lower misconduct, no incompatible moves and high rates of alternative resolutions. A prospective study using more extensive prisoner and staff interviews and unit observation might better confirm the effectiveness of the community as method activity within the TC’s, as well as prisoner and staff views on if and why behaviors changed as a result of TC involvement. Comparisons with inmate interviews in general population might give a more accurate comparison. Is the prisoner-correctional officer relationship truly different? These are all worthwhile questions to pursue.

The findings of this study indicate that alternative resolutions can be used at a fairly substantial rate within a correctional facility and supports the generally positive research outcomes associated with therapeutic communities. Future research is recommended on how to best use alternative resolutions in prisons and to better understand how and why therapeutic communities work.

## Notes

1. Direct supervision emphasizes the importance of interaction and relationship building with prisoners. Correctional officers are expected to utilize personal influence more than coercion in gaining compliance from prisoners. While associated with newer, more open prison architecture, direct supervision can be utilized in a variety of custodial settings (Wener, 2006).

2. The variance ratio is the ratio of the standard deviation to the arithmetic mean, *s/*x. The closer the ratio comes out to 1.0 (large *s* relative to the mean) the greater the variability in the data, suggesting a skewed distribution. Greater variability makes it harder to make confident sample estimates unless either the samples and/or mean differences are large.
